# Thank you to all of our reviewers in 2019

**DOI:** 10.1038/s42003-020-0782-y

**Published:** 2020-02-10

## Abstract

2530 individual reviewers contributed to the peer review process at *Communications Biology* in 2019. We acknowledge and thank each of them here.

In its second full year as a journal, *Communications Biology* received 1642 research article submissions, of which 868 were sent for external review. Each of the 2530 individuals who gave their time to ensure a fair and thorough peer review process are recognized below in alphabetical order by surname. We are truly grateful to all of our reviewers. Journal staff take full responsibility for any misspellings, omissions, or other errors.

We are truly grateful to all of our reviewers

**Aarestrup—Amos**

Kim Aarestrup

Omar Abdel-Wahab

Reza Abdi

Atefeh Abdolmanafi

Takaai Abe

Oscar John Abilez

Giancarlo Abis

Julien Ablain

Eyal Abraham

Jakub Abramson

Enas Abu Shah

Federica Accornero

Maegen Ackermann Borzok

Steven Adie

Neeraj Agarwal

Eneritz Agirre

Niaz Ahmad

Tae-Hyuk Ahn

Nicole Aiello

Hideki Aihara

Makoto Akashi

Yasuto Akiyama

Aleksei Aksimentiev

Artemis Aktipis

Sk. Kayum Alam

Balbino Alarcón

Paul Albert

Jacqueline Alblas

Philippe Albouy

Michaeline Albright

Joseph Alcorn

Mark Alkema

Kristina Allen-Brady

Benjamin Allen

Charles Allen

Luke Allsopp

Alejandra del Carmen Alonso

Donát Alpár

J. Andrew Alspaugh

Miguel Sánchez Álvarez

Kaushalya Amarasinghe

Jaime Amengual

Ali Amin-Mansour

Daniel Amor

William Amos

**An—Azvedo**

Yu An

Bela Anand-Apte

Bogi Andersen

Mark Anderson

Sarah Anderson

Massimiliano Andreazzoli

Adrian-Ştefan Andrei

Andrews Andrew

Matthew Andrews

Takashi Angata

Fernando Anjos-Afonso

David Ann

Lindsey Anstine

Jonathan Antcliffe

Bruno Antonny

Demet Arac

Manuel Aranda

Joseph Arboleda-Velasquez

Rafal Archacki

Nathan Archer

P. Alexander Arguello

Hiroyoshi Ariga

Shin-ichi Arimura

Kenton Arkill

Dan Arking

E. Virginia Armbrust

Maickel Armenteros

David Armstrong

Steven Artandi

Ryoichi Asaka

Ryutaro Asano

Lorna Ashton

Philip Ashton

Florencia Assaneo

Jeffrey Atkinson

Haruyuki Atomi

Scott Atwood

Nadia Aubin-Horth

Marc Louis Augé

Yurii Aulchenko

Tim Aumann

Mikhail Avdeev

Taj Azarian

Herlander Azevedo

**Baarends—Bayat**

Willy Baarends

Leslie Babonis

Jaume Bacardit

Annabell Bachem

Thierry Backeljau

Tudor Badea

Christian Badr

Robert Bagchi

Martin Iain Bahl

Yong-Sun Bahn

Scott Bailey

Calum Bain

Avinash Bajaj

Ambroise Baker

Suzanne Baker

Nuredin Bakhtiari

Michal Balberg

Miklós Bálint

Justin Balko

Thomas Balle

Elizabeth Balmayor

David Baltrus

Takeshi Bamba

Laura Banaszynski

Peter Bandettini

Anirban Banerjee

Florence Bansept

Markus Bantscheff

Debmalya Barh

Michael Barker

Timothy Barnett

Nicolas Barnich

Rodolphe Barrangou

Elias Barriga

Madelaine Bartlett

Thomas Bartnikas

Rachael Bashford-Rogers

Pouya Bashivan

Patricia Bassereau

Oliver Bathe

Alexandra Battaglia-Mayer

Frederic Batteux

Nicholas Bauman

Florian Baumgart

Tobias Baumgart

Isabel Bäurle

Krutika Bavishi

Mohammad Bayat

**Beauregard—Bitler**

Pascale Beauregard

Oren Becher

David Beck

Sarah Beck

Stephen Beckett

Gregg Beckham

Sandra Beer-Hammer

Peter Beernink

Jean-Claude Béïque

Shai Bel

Nicholas Bello

Bertrand Benazeraf

Fabio Benedetti

Alexandre Emmanuel Benedetto

Michael James Benton

Beben Benyamin

Nicolas Berbari

Monica Berdugo

Igor Berezovsky

Frederic Berger

Michael Berger

Til Ole Bergmann

Mónica Berjón-Otero

Ben Berks

Gilbert Bernier

David Bernlohr

Fabrice Bertile

Giulia Bertolin

Francesco Bertolini

Christian Beste

Sven Bestmann

Doron Betel

Krishna Bhat

Tripta Bhatia

Mallar Bhattacharya

Akshay Bhinge

Jin-Song Bian

Jake Emmerson Bicknell

Gavin Bidelman

Jan Bieschke

Tamir Biezuner

David Bikard

Jose Ramon Bilbao

Silvija Bilokapic

Albrecht Bindereif

Alexander James Roy Bishop

Alexandre Bisson

Benjamin Bitler

**Bjarnholt—Brochado**

Nanna Bjarnholt

Craig Blackstone

Jeffrey Lawrence Blanchard

William Blaner

Bradley Blaser

Stephanie Bleicken

Ran Blekhman

Arnold Bloom

Kyle Blum

Bruce Bochner

Joël Bockaert

Robert Bodizs

Matthias Boer

Scott Boitano

Yves Bollen

Ben Bond-Lamberty

Vincent Bonhomme

Titouan Bonnot

Marielle Boonen

Thomas Borch

Mark Borden

Nica Borgese

Jan Born

Steven Bosinger

Alberto Bosque

Jan Bosteels

Mihnea Bostina

Bettina Böttcher

Pavel Bouchal

William Bourguet

Luc Bousset

Vassiliki Boussiotis

Than James Boves

Vladimir Boza

Thomas Bozza

Rogier Braakman

Ian Bradbury

Anindita Brahma

Simon Johannes Brandl

Martin Brandt

Jochen Braun

Timothy Paul Brawn

Michael Breakspear

Martin Brechbiel

Gerome Breen

Emery Bresnick

Warwick Britton

Santiago Brizuela

Ana-Rita Brochado

**Broens—Bybee**

Els Broens

Johannes Broichhagen

Benjamin Brooks

Charles Brooks

Mark Broom

Mikael Brosché

Christopher Brown

David Brown

James Brown

Nick Brown

Susan Brown

William Brown

Hannah Bruce Macdonald

Mark Brynildsen

Alastair Buchan

Susan Buchanan

Johannes Buchner

Evgeny Budygin

Alexander Buell

Francis David Bulbeck

Serdar Bulun

Moritz Bunemann

Silvia Bunge

Stephen Buratowski

Pamela Burger

Nicola Burgess-Brown

Kevin Burgess

Brianne Burkinshaw

Alessia Buscaino

Eduardo Esteban Bustamante

Orpheus Michael Butler

Lucie Buttay

Manish Butte

Joel Buxbaum

Seth Bybee

**Cabernard—Chang**

Clemens Cabernard

Israel Cabeza de Vaca

Lisa Cabrita

Arnaud Cachia

Hongmin Cai

Shi-Qing Cai

Alison Callahan

John Callan

Eva María Camacho

Alexander David Cameron

Michael Campana

Javier Campos-Gomez

Turhan Canli

Cinzia Cantacessi

Elizabeth Canty-Laird

Gang Cao

Song Cao

Yihai Cao

Daiana Capdevila

Yossi Capua

Michele Carbone

Silvia Cardona

Tanai Cardona

Giuseppina Caretti

Lelis Antonio Carlos-Junior

Jeremy Carlton

Guido Carpino

James Carson

Gabriella Caruso

Beatriz Carvalho

Wayne Carver

Greg Cary

Fernanda Casares

Patrick Casey

Neil Cashman

Svenja Caspers

Gladys Iliana Cassab

Guillermo Cassini

Joaquín Castilla

Carmen Castresana

Cesar Castro

Byron Caughey

Thomas Cech

Jonathan Cedernaes

Laura Cendron

Giulio Cerullo

Barbaros Cetin

Hirak Chakraborty

Anil Kumar Challa

Jeffrey Chambers

Cliburn Chan

David Chan

Kung-Sik Chan

Chawnshang Chang

Eric I-Chao Chang

Matthew Chang

Nan-Shan Chang

Veronica Chang

Wakam Chang

Yu-Lin Chang

**Chapiro—Chun**

Grigori Chapiro

Craig Chapman

John Chappell

Konstantinos Chatzistergos

V. Bala Chaudhary

Sylvain Chauvette

Caiyan Chen

Cheng-Yao Chen

Chi-Hua Chen

Guo-Qiang Chen

Hangzi Chen

Hannah Chen

Huizhong Chen

Jiekai Chen

Jing Chen

Joseph Chen

Kai-En Chen

Liangbiao Chen

Ling-Ling Chen

Yibing Chén

Peng Chen

Qi-Jun Chen

Qing Chen

Rujin Chen

Shanshuang Chen

Shuibing Chen

Ting Chen

Xiaowu Chen

Yang Chen

Yingchuan Chen

Z. Jeffrey Chen

Zhe Chen

Zheng Chen

Zi Chen

Bo Cheng

Daojun Cheng

Hai-Ying Mary Cheng

Keith Cheng

Wei Cheng

Xiu-Tang Cheng

Vikram Chhatre

David Chia

Kevin Childs

Marco Chilosi

Fernando Chirdo

Shadreck Chirikure

Magdalena Chmiela

Mordechai Choder

James Chodosh

Hee-Jung Choi

Se Hoon Choi

Hélène Choquet

Antonios Christou

Jonathan Chubb

Sung Chun

**Chung—Czech**

Ren-Hua Chung

Marco Cianchetti-Benedetti

Pietro Cicuta

Chiara Cilibrasi

Peter Cistulli

Matthew Clapham

Amanda Clare

John Clarke

Stephen Clarke

Marcus Clauss

Kendall Clements

Thomas Clements

Hans Clevers

Cristian Coarfa

Brian Cobb

Brian Codding

Ruben Coen-Cagli

Tom Coenye

Cyrille Cohen

Monica Colaiacovo

Allen Collins

Catherine Collins

Celine Colnot

Marco Colonna

Francesco Colucci

Karen Colwill

Iñaki Comas

Liza Comita

Katherine Conen

Marion Coolen

Jan Cools

Scott Cooper

Luc Cornet

Benjamin Corona

Jill Corre

Rebecca Corrigan

Rondald Cortright

Mehmet Ilyas Cosacak

Stephen Cose

Andre Costa da Silva

Mark Costello

Jaeda Coutinho-Budd

Christopher William Cowan

Robert Cramer

Brian Crane

Matthew Crane

Sylvie Crasquin

Nicole Creanza

Rhiannon Creasey

Alessandro Cresci

James Crowe

Perrine Cruaud

José Cuezva

Bianxiao Cui

Haitao Cui

Jianmin Cui

Chris Cunningham

Laura Czech

**D’Adda—Del Amo**

Fabrizio D'Adda di Fagagna

Isabella D'Ambra

Matteo D'Antonio

Maurizio D'Antonio

Alessandra D'Azzo

Humberto D'Muniz Pereira

Jacek Dabert

Rafael Daga

Onur Dagliyan

Ingrid Dahlman

Jungui Dai

Lisheng Dai

Zhongmin Dai

Ayoub Daliri

Kevin Gerard Daly

Paola Dama

Alejandro Damian Serrano

Hien Dang

Charlier Daniel

Joe Daniele

Jens Danielsson

Cira Dansokho

Thomas Daubon

Liliana Dávalos

Emily Davenport

Stephen David

Yael David

Shawn Davidson

Jon Day

Kenneth De Baets

Sietse de Boer

Guillermo de Carcer

Geoffrey de Couto

Dirk de Graaf

Primal de Lanerolle

Irene de Lázaro

Alex de Marco

Gonzalo de Prat-Gay

Amanda de Souza

Paulo De Souza

Diego De Stefani

Wim de Vries

Douglas Dean III

Kevin Michael Dean

Don DeAngelis

Joris Deelen

Tony DeFalco

Eva Maria Del Amo

**Del Greco—Dzierzak**

Fabiola Cristina Del Greco

Miguel Angel Del Pozo Barriuso

Borja Del Pozo-Cruz

Luiz-Eduardo Del-Bem

Pierre-Marc Delaux

Henri-Jacques Delecluse

Peyman Delparastan

Jerome Delroisse

Dmitri Demidov

Dawid Deneka

Qiaolin Deng

Ashok Deniz

Atul Deshmukh

Riddhi Deshmukh

Anne Deslattes Mays

Paula Desplats

Marie-Ange Deugnier

Jörg Deutzmann

Saravana Mohan Dhanasekaran

Ali Dhinojwala

Antonio Di Franco

Monica Di Luca

Antonio Di Sabatino

Dolores Di Vizio

Kamran Diba

James DiCarlo

Sebastian Diecke

Susanne Diekelmann

Stephen Paul DiFazio

Bauke Dijkstra

Panayiotis Dimitrakopoulos

Rumiana Dimova

Li Ding

Aaron Dinner

Phat Vinh Dip

Ottavia Dipasquale

Scott Dixon

Ron Do

Katharina Dobs

Chris Dockendorff

Antony Dodd

James Dodge

Hideyuki Doi

Nikolay Dokholyan

Patrick Dolan

Andrey Dolgov

Daniel Dominguez

Changjiang Dong

Yan Dong

Steven Dowdy

George Dragoi

Mikhail Drobizhev

Zhen-Yu Du

Zhenglin Du

Raymond DuBois

Anna Dubrovska

Timothy Dubuc

Jorge Duconge

Olga Dudchenko

Eric Dufresne

Amanda Dugan

Priya Duggal

Reuven Dukas

Nicholas Dulvy

Jason Dunlop

Alex Dunn

Michael Dunn

Warwick Dunn

Beatrice Durand

Elaine Dzierzak

**E**

Alice Easton

Hariharan Easwaran

Darius Ebrahimi-Fakhari

Mo Ebrahimkhani

Anastassios Economou

Frank Edenhofer

Matthew Edgington

Christine Edwards

Thomas Edwards

Britta Eickholt

Paul Ekert

Samir El-Andaloussi

Satheesh Elangovan

Jean-François Eléouët

Natalie Elia

Kirsten Ellegaard

Felix Ellett

David Anthony Elliott

Patrick Emery

David Emms

Scott David Emr

Abdul-Hamid Emwas

Toshiya Endo

Jutta Engel

Christine Engeland

Tobias Engl

David Engman

Daniel Enosi Tuipulotu

Vanessa Era

Ari Ercole

Jeffrey Erickson

Jason Ernst

Oliver Ernst

Ali Ertürk

Terry Eskenazi

Olivier Espéli

Cesar Ignacio Espinoza

Carlos Estella

Borja Esteve-Altava

Iryna Ethell

Manuel Etzkorn

Aymerick Eudes

Michelle Evans

Yoichi Ezura

**Fabre—Flórez**

Emmanuelle Fabre

Luca Fabris

Oliver Fackler

jamila Faivre

Pascal Falter-Braun

Damilare Famakinde

Kelong Fan

Longhou Fang

Yue Fang

Donna Farber

Steven Farber

William Farnaby

Paul Farrell

Rhys Farrer

Reinhard Fässler

Amos Fatokun

Christine Faulkner

Kenan Fears

Leonardo Manir Feitosa

Baomin Feng

Fleur Ferguson

Ricardo Fernandes

Martin Fernandez-Zapico

Alisdair Fernie

Sebastien Ferreira-Cerca

Manuel Ferrer

David Ferrier

Mark Ferris

Andrzej Fertala

William Festuccia

Daniel Field

Andrew Fields

Milos Filipovic

Massimo Filippi

Christine Fillmore Brainson

Gottfried Fischer

Harold Fisk

David FitzGerald

Julia Catherine Fitzgerald

Angeleen Fleming

Melanie Sarah Flint

Hauke Flores

Laura Flórez

**Fon—Furukawa**

Edward Fon

Stephen Fong

Vera Fonseca

Juan Carlos Fontecilla-Camps

Beatriz Fontoura

Stuart Forbes

Talitha Ford

Federico Forneris

Jannine Forst

Leonard Foster

Dimitrios Fotiadis

Nicholas Foulkes

Chris Foulon

Aaron Fox

Marco Aurélio Gallo de França

Nora Franceschini

Diego Franco

Nikolaos Frangogiannis

Susanne Franssen

Lori Frappier

Lauchlan Fraser

Andrew French

Serita Frey

Kevin Friedland

Jonathan Friedman

Seth Frietze

Keith Jordan Fritzsching

Dong-Jing Fu

Tian-Ming Fu

Cesar de la Fuente

Yuichi Fujita

Akiyoshi Fukamizu

Tomokazu Fukuda

Nobuyuki Fukushima

Ben Fulcher

Deborah Fuller

Yoshiaki Furukawa

**Galanty—Glubb**

Yaron Galanty

Gabriel Galea

Mary Galinski

Peter Gallant

Marco Gallio

Imed-Eddine Gallouzi

Benoit Gamain

Eric Gamazon

Karuna Ganesh

Haichun Gao

Shaorong Gao

Laurent Gapin

Michael Garabedian

Pilar Garcia-Jimenez

Claudio Araya Garcia

Paul Garcia

Andrew Gardner

George Garinis

Pere Garriga Sole

John Garthwaite

Fabio Gasparini

Eric Gaucher

Patrick Gauthier

Arnaud Gautier

Rasheed Gbadegesin

Tian Ge

Paul Geha

Peter Geldhof

Martin Genner

George Gentsch

Paul George

Malte Gersch

Kristina Gervin

Benjamin Gewurz

Sina Ghaemmaghami

Michael Giacomelli

Alasdair Gibb

Régis Giet

Tristan Gilet

Christian Gilissen

Fiona Ginty

Teresa Giraldez

Pierre Gladieux

David Glanzman

Leon Glass

Clemens Glaubitz

Benjamin Glick

Joseph Glorioso

David Ryan Glowacki

Elisabeth Glowatzki

Dylan Glubb

**Gobet—Grivennikov**

Angélique Gobet

Susana Godinho

Chandraiah Godugu

Jacky Goetz

Rajan Gogna

Nadine Gogolla

Paul Gold

Matias Goldin

Agnieszka Aleksandra Golicz

Sandra Gollnick

Nardhy Gomez-Lopez

Natalia Gomez-Ospina

Karina Gómez

Roger Gomis

Carla Goncalves

Kevin Andrew Uy Gonzales

Patrick Rolland Gonzales

Sara Gonzalez-Arranz

Alicia Gonzalez

Cayetano Gonzalez

Kaundinya Gopinath

Tobias Goris

Jorg Goronzy

Eric Gorscak

Christian Gortázar

Magdalena Gotz

Aymeric Goyer

Jesse Goyette

Peter Gräber

Claudio Grandi

Jennifer Grandis

Valerie Grandjean

Jorg Grandl

Carsten Grashoff

David Grayden

Shane Grealish

Alissa Greenwald

Victor Greiff

Christina Gremel

Alex Greninger

Adam Grieve

Robert Griffin-Nolan

Patrick Griffin

Josh Griffiths

Kalanit Grill-Spector

Brock Grill

Sergei Grivennikov

**Grobner—Guzman**

Gerhard Grobner

Gideon Grogan

Ursula Grohmann

Michael Groll

Corrinne Grover

Marie Luise Grünbein

Hajo Grundmann

Thomas Grunt

Chenghua Gu

Qingdong Guan

Yafeng Guan

Michael Guarnieri

Olafur Gudmundsson

Xavier Guell

Jeremy Guggenheim

Thomas Guillerme

Josee Guirouilh-Barbat

Babett Günther

Changmiao Guo

Feng Guo

Hwai-Chen Guo

Xing Guo

Yongfeng Guo

Rani Gupta

Vsevolod Gurevich

Yaron Gurovich

Kiran Gurung

Mika Gustafsson

Anna-Karin Gustavsson

Valeria Guzman-Luna

**Haehn—Hedrich**

Daniel Haehn

Emily Haeuser

Ziad Hafed

Carsten Hagemann

Katsumi Hagita

Matthew William Hahn

Jens Hahne

Stefan Hallermann

Liberty Hamilton

Michael Hammerling

Dillon Jack Hammill

Jinju Han

Leng Han

Bertrand Hankoua

Roger Hanlon

Christian Hansel

Diana Hansen

Kiah Hardcastle

Katarzyna Harezlak

Martin Häring

Alex Harkess

Michael Jonathan Harms

William Harnett

Lea Harrington

Tony Harris

David Hart

Traver Hart

Adam Hartstone-Rose

Tetsuya Haruyama

Alan Harvey

Natasha Harvey

Wendy Harwood

Kenji Hashimoto

Henrik Hasman

Seyed Hasnain

Haralambos Hatzikirou

Carolin Haug

Melissa Hawkins

Oliver Haworth

Michael Hawrylycz

Yohei Hayashi

Gerald Hazelbauer

Roni Hazim

Guangming He

Jian He

Mei He

Drew Headley

William Heath

Nemat Hedayat-Evrigh

Rainer Hedrich

**Heffern—Holstein**

Marie Heffern

Noel Heim

Louise Heinrich

Carl-Philipp Heisenberg

Maija Iris Heller

Klaas Hellingwerf

Ute Hellmich

Glyn Hemsworth

Isabelle Henry

Melissa Herbst-Kralovetz

Miles Herkenham

Meenhard Herlyn

Juan Hermoso

Uri Hertz

Erik Herzog

Claudio Hetz

Andreas Heuer

Thierry Heulin

Derren Heyes

John Heymach

Margaret Hibbs

Heather Hickman

Ville Hietakangas

Shu Hui Hiew

Matt Higgins

Falk Hildebrand

William Hildebrand

Kiyoshi Hirahara

Noriko Hiroi

Kendal Hirschi

Matthew David Hitchings

Timothy Hla

Kwo Wei David Ho

Linda Hoang

Angela Hodges

Alan Hodgkinson

Leanne Hodson

Jason Hoeksema

Luke Hoeppner

Jules Hoffmann

Thomas Hoffmann

Johan Hofkens

Ralf Hofmann

Ctirad Hofr

Martin Högbom

Steven Holen

Linda Holland

Wolfgang Holnthoner

Nicholas Holowka

Thomas Holstein

**Holzapfel—Hytonen**

Gerhard Holzapfel

Bernhard Hommel

Chung Chau Hon

Angelo Hooker

Richard Hooley

Amanda Hopes

Samantha Hopkins

Adam Hoppe

Mathew Horrocks

Naoto Hoshi

Thomas Hossie

Michael Hothorn

Dennis Hourcade

Kalina Hristova

Ming Hsu

Bing Hu

Dehui Hu

Gangqing Hu

Jim Hu

Jinghua Hu

Zheng Hu

Baokang Huang

Chi-Hsien Huang

Jiyue Huang

Qixing Huang

Shuang-Quan Huang

Tengbo Huang

Wen Huang

Yanyi Huang

Yifei Huang

Maite Huarte

Peter Hubka

Gregory Hudalla

Pablo Huertas

Mark Hughes

Zoe Hughes

Kam Man Hui

Benjamin Hume

S. Perwez Hussain

Andrew Hutchins

Gereon Hüttmann

Mikko Huttunen

Jennifer Huynh

Vincent Hyenne

Vesa Hytonen

**I**

Sherif Abdelaziz Ibrahim

Dora Il'yasova

Kiho Im

Leah Imlay

Salvatore Incontro

Stefano Indraccolo

Keiichi Inoue

Marco Invernizzi

Grzegorz Ira

Iker Irisarri

Rossitza Irobalieva

Nicholas Irwin

Tadashi Isa

Maria Isaguliants

Atsushi Ishida

Shin’ichi Ishiwata

Leyla Isik

Taichi Isobe

Raffaella Isola

William Israelsen

Michelle Itano

Yuji Ito

Plamen Ivanov

Lionel Ivashkiv

Aishwariya Iyengar

Sandro Carvalho Izidoro

**J**

Stephen Jackson

Ralf Jacob

Jonathan Jacobs

David Jacobson

Mehdi Jafarnejad

Jacob Jaffe

Rakesh Jain

Young Charles Jang

Gert Jansen

jean-michel Jault

Jean-Marc Jeckelmann

Christopher Jeffrey

Matlock Jeffries

Michael Jenkins

Michael Jennions

Mogens Jensen

Poul Jensen

Thomas Jensen

Sissel Jentoft

Taeck Joong Jeon

Rolf Jessberger

Guang Ji

Zhengping Jia

Huifeng Jiang

Lin Jiang

Liwen Jiang

Peng Jiang

Xiao-Tao Jiang

Xiaohong Jiang

Jing Bo Jin

Johann Joets

Hanna Katarina Lilith Johansson

Linda Johansson

Simona John von Freyend

Erik Johnson

Michael Johnson

Peter Johnson

Shannon Lyn Johnson

Mohit Kumar Jolly

Peter Jonas

Rose Jones

Stephanie Jones

Daniel Jordan

Peter Jordan

Nitin Joshi

Hsueh-Fen Juan

Nathalie Juge

Jean-Philippe Julien

Jae-Young Jung

Parijat Juvale

**Kacsoh—Khor**

Balint Kacsoh

Kohmei Kadowaki

maiko kagami

Igor Kagan

Itamar Kahn

Eirini Kaiserli

Chikara Kaito

Alexandr Kalinin

Tomer Kalisky

Charalampos Babis Kalodimos

Bozena Kaminska

Clemens Kaminski

Jian-Sheng Kang

Le Kang

Radoslava Kanianska

Aphrodite Kapurniotu

Siddhartha Kar

Ragnhildur Thora Karadottir

Ioannis Karakikes

Canan Karakoç

Mehmet Erman Karasu

Maria Karayiorgou

Lamya Karim

Georgia Karpathiou

Justine Karst

Kenji Kasai

Maria Kasper

Takenobu Katagiri

Benita Katzenellenbogen

Manabu Kawada

Brian Kay

Hajime Kayanne

Patrick Keeling

Kylene Kehn-Hall

Almut Kelber

Peter Kele

Erin Kelleher

Laurent Keller

Neil Kelley

Thomas Kelley

Jack Kelly

Ian Kelsall

Candia Kenific

Brian Kennedy

Alex Kentsis

Tijs Ketelaar

Mireille Khacho

Philipp Khaitovich

Walid Khaled

Chiea Chuen Khor

**Khurana—Komor**

Leepakshi Khurana

Cezar Khursigara

Jeff Kidd

Claudine Kieda

Claudine Marie-Therese Kieda

Florian Kiefer

Nicolas Kieffer

Tim Kietzmann

Yoshitomo Kikuchi

Bong-Soo Kim

Jeong-Il Kim

Junhwan Kim

Oanh Thi Phuong Kim

Taewoo Kim

Yongsoo Kim

Young Tae Kim

Declan King

John King

Chrissa Kioussi

Girish Kirimanjeswara

Raj Kishore

Daiju Kitagawa

Andrew Kitchener

Shinae Kizaka-Kondoh

Jonathan Klassen

Ramon Klein Geltink

Benjamin Peter Kleinstiver

Paul Klenerman

Karl Klose

Jerome Kluza

Andrey Klymchenko

Angela Knapp

Allison Knoll

Keith Knutson

Jaewon Ko

Jay Ko

Henner Koch

Richard Kock

Ullasa Kodandaramaiah

Michael Koeppen

Gou Young Koh

Kyunghee Koh

Douglas Kojetin

Shihoko Kojima

Vasilis Kokkoris

Denis Kolbasov

Stanislav Kolesnikov

Evangelos Kolettas

Alexis Komor

**Kondrashova—Kyprianou**

Olga Kondrashova

Xiangjuin Kong

Talia Konkle

Maria Kontaridis

Aikaterini Kontrogianni-Konstantopoulos

Frank Kooy

Michael Kormann

Sigrun Isolde Korsching

Zsombor Koszegi

Tomotsugu Koyama

Michael Kracht

Alwin Kraemer

Natalie Krahmer

Natalie Krahmer

Sven Alexander Kranz

Mark Krebs

Matthias Kreuzer

Anja Krieger-Liszkay

Lasse Kristoffer Bak

Sergei Krivov

Anders Krogh

Andrea Krueger

Sergey Krupenko

Karsten Kruse

Takanori Kubo

Yoshihiro Kubo

Sara Kuebbing

Martin Kuhlwilm

Waldemar Kulig

Amit Kumar

Arvind Kumar

Sudhir Kumar

Sudha Kumari

Krushnamegh Kunte

Comert Kural

Harley Takatsuna Kurata

Leo Kurian

Daisuke Kurihara

Peter Kurre

Sebastian Kurscheid

Soo-Heon Kwak

Natasa Kyprianou

**LaBaer—Lei**

Joshua LaBaer

Adam Lacy-Hulbert

Michael Robert Ladomery

Anne Lagendijk

Juha Lahnakoski

Jinsheng Lai

Nan Laird

Ryan LaLumiere

John LaMattina

Katja Antoinette Lamia

Peter Lammers

Fei Lan

Erin Landguth

Michael Landy

Vinzenz Lange

Tobias Langenhan

Lucia Languino

Michael Lanzer

Audrone Lapinaite

Carolyn Larabell

Maximilian Larena

Janine LaSalle

Paul Lasko

Thomas Lasko

Emanuele Claudio Latagliata

Peter Latham

Richard Lathe

Justin Lathia

Hakwan Lau

Patrick Laufs

Gregory Lavieu

Michelle Lawing

Yann Le Guen

Corentin Le Magueresse

Antoine Le Quere

Elizabeth le Roux

David Lea-Smith

Katie Leach

Francesca Leasi

Sarah Lebeis

Claire Leblond

Maël Lebreton

Carlito Bangeles Lebrilla

Bernett Teck Kwong Lee

Bongsoo Lee

Esther Lee

Hunjoong Lee

Jung-Shin Lee

Kyung-Tae Lee

Kyuwan Lee

Sang Jin Lee

Tsung-Ming Lee

Roger Lefort

Christian Lehmann

Ben Lehner

Qunying Lei

**Leibold—Liman**

Josef Leibold

Alexander Leitner

Stéphane Lemaire

Jacob Lemieux

Jiapeng Leng

Birgit Lengerer

Heinz-Josef Lenz

Livia Leoni

Konstantin Lepikhov

Juan Lerma

Tommy Ling Fong Leung

Romain Levayer

Gabriel Leventhal

Francis Levi

Gustavo María Levrino

Randolph Lewis

Mathew Lewsey

Bin Li

Chao Li

Dong Li

Fu-Shuang Li

Howard Li

Jian Li

Jianguo Li

Jianing Li

Jingyi Jessica Li

Lei Li

Mingyue Li

Peng Li

Pengfei Li

Pingwei Li

Qiang Li

Rong Li

Tuo Li

Xiaoxia Li

Xinzhong Li

Yan Li

Yanchuan Li

Yang Li

Yuhui Li

Zhen Li

Jennifer Liang

Liya Liang

Wenxing Liang

James Liao

Jian-Xiang Liao

Pablo Librado

Jonathan Licht

Mathias Lichterfeld

Lenine Liebengerg

Thor-Seng Liew

Catherine Lilley

Andrew Lim

Chai Lim

Lee-Sim Lim

Sai-Kiang Lim

Theam Soon Lim

Santiago Lima

Emily Ruth Liman

**Lin—Lytton**

Haotian Lin

Jer-Young Lin

Jiandie Lin

Meng Lin

Michael Lin

Matthew Lincoln

Peter Lindblad

Maurine Linder

Annie Lindgren

Wenhua Ling

Joachim Lingner

Jonathan Lippiat

Tim Littlewood

Yael Litvak

Anastasia Litvintseva

Bao Liu

Caigang Liu

Guei-Sheung Liu

Haiguang Liu

Hao Liu

Jin Liu

Jinqiang Liu

Pengyuan Liu

Qi Liu

Ruisheng Liu

Thomas Liu

Xiangguo Liu

Zhijie Liu

Andy LiWang

Matthew David Lloyd

Stephanie Lo

Ken Locey

Mark Loewen

Samatha Loh

Ingrid Lohmann

Ivan Lomakin

Giovanna Lombardi

Michael Longaker

Mart Loog

Alizee Lopez-Persem

Margarita Lopez-Uribe

Alberto Lopez

Antoine Loquet

Yonggen Lou

Jonathan Lovell

Sara Lovisa

Jian Lu

Wei Lu

Elizabeth Lucas

Paul john Lucassen

Karolin Luger

Leo Lui

Jan Lünemann

Chongyuan Luo

Jian-Hua Luo

Ray Luo

Ruibang Luo

Paolo Luschi

David Lyons

Mark Lyte

William Lytton

**Ma—Marson**

Hongshen Ma

Jian Ma

Jianjie Ma

Li-Jun Ma

Qingjun Ma

Yu-qiang Ma

Ben Daniel MacArthur

Lara Macheriotou

Sara Macias

Sonya MacParland

Dan MacQueen

Alessandra Magistrato

Giovanni Maglia

Finlay Maguire

Magdy Mahfouz

Sabine Mai

Ana Teresa Maia

Susannah Maidment

Alexander Maier

Timm Maier

Bernardo Mainou

Tomoko Makishima

Carolina Makowski

Leonel Malacrida

Alizee Malnoe

Alexander Malogolovkin

Gabriel Malouf

Rafael Manez

Fabio Manfredini

Aswin Mangerich

Thomas Manke

Paul Manley

Ana Maria Manso

Jose Manso

Sara Mantero

Kelly Manthei

Xiuguang Mao

Marina Mapelli

Vincent Marconi

James Marden

Thomas Maresca

Andriana Margariti

Jan Marienhagen

Julieta Marino

Riccardo Marioni

Ines Marques

Petra Marschner

Jennifer Marsh

Alexander Marson

**Martin—Mertz**

Adam Christopher Martin

Brent Martin

Donna Martin

Rene Martin

T. John Martin

William Martin

Marina Martinez-Garcia

German Martinez

Ludmila Martínková

Andreas Marx

Asier Marzo

Stefania Masci

Ameya Mashruwala

Patrick Masson

Maria Grazia Masucci

Naoki Masuda

Carlos Mata

Ulrike Mathesius

Nobuyuki Matoba

Michael Matschiner

Ikki Matsuda

Takashi Matsuo

Daumantas Matulis

Andrew Maurer

Satyajit Mayor

Yuval Mazor

Elizabeth McCarthy

Timothy McClintock

Alistair McCormick

Edwina McGlinn

Sheena McGowan

Leeanne McGurk

Charles McKenna

Gian-Luca McLelland

Michael McManus

Katelyn McNair

Sean Meaden

Jason Mears

Fleur Meddens

Hamish Meffin

Akira Meguro

Devang Mehta

Samuel Meier-Menches

Fanju Meng

Fanwei Meng

Jia Meng

Wei Meng

Weihua Meng

Hugo Merchant Nancy

Arthur Mercurio

Taha Merghoub

Jean-Louis Mergny

Maarten Merkx

Janet Mertz

**Meunier—Morikawa**

Nicolas Meunier

Rui Miao

Catherine Michaux

Guy Franklin Midgley

Michihiro Mieda

Alexander Mildner

Lee Miller

Maximilian Miller

Yury Miller

Carsten Mim

Cyrus Mintz

Seyed Abolghasem Mirroshandel

Ali Miserez

Masanori Mishima

Karolis Misiunas

Christina Mitchell

Nora Mitchell

Diana Mitrea

Alissa Mittnik

Keiichi Mochida

Nina Moderau

Mauro Modesti

Francie Moehring

Ashley Moffett

Andreas Möller

Vito Monaco

William Montevecchi

Grant Montgomery

Cedric Montigny

Joseph Montoya

Emily Moore

John Moore

Marco Moracci

Martin Morad

Trevor Francis Moraes

Violaine Moreau

José María Moreno-Navarrete

Karen Moreno

Michael Morgan

Rhys Morgan

Branden Scott Moriarity

Masato Morikawa

**Morimoto—Mykytyn**

Jumpei Morimoto

Yusuke Morimoto

Yasu Morita

Valentina Moro

Toshiro Moroishi

Maurizio Morri

Ciaran Morrison

Ala Moshiri

Sergei Moshkovskii

Allan McI Mowat

David Mueller

Elisabetta Mueller

Eugene Mueller

Debabrata Mukhopadhyay

Saikat Mukhopadhyay

Vijaykumar Muley

Florian Muller

Lyle Muller

Timo Müller

Steven Munger

Estela Muñoz

Yasuteru Muragaki

Ryutaro Murakami

Walter Lee Murfee

Cormac Murphy

Shauna Murray

Dhaarini Murugan

Gabriella Musacchia

Giovanna Musco

Jacob Musser

Aline Muyle

Cameron Myhrvold

Kirk Mykytyn

**N**

Takuro Nakagawa

Masatoshi Nakajima

Masanori Nakayama

Yunjun Nam

Jagdeep Nanchahal

Jayakrishnan Nandakumar

Shivangi Nangia

Arshan Nasir

Guillermo Navalon

Stephen Neely

David Roy Nelson

Jeanne Nerbonne

Donna Neuberg

Elsa Neubert

Philipp Neudecker

Harald Neumann

Simon Neumann

Jiri Neuzil

Michael Newton

Philip Ng

Liem Hieu Nguyen

Guangjian Ni

Joseph Nickels

Dariusz Niedzwiedzki

Morten Nielsen

Peter Nielsen

Yusuke Niino

Evgeni Nikolaev

Viacheslav Nikolaev

Eleni Nikolakaki

IngMarie Nilsson

Guogui Ning

Ellen Nisbet

Hiroshi Nishimaru

Wataru Nishimura

Yasuhiko Nishioka

Lee Niswander

Kechang Niu

Wei Niu

Hitoshi Niwa

Maho Niwa

Krishna Niyogi

Wendy Noble

Jean-Paul Noel

Przemyslaw Nogly

Javad Noorbakhsh

Fernando Novas

Laura Novellasdemunt

Dorota Nowak

Alessandro Nucara

Keiji Numata

Dmitri Nusinow

**O**

Joan O'Brien

Cecilia O'Leary

Michelle O’Malley

Steven O’Reilly

Desmond Oathes

Johannes Oberwinkler

Kentaro Oh-hashi

Yuko Ohara-Nemoto

Shigeo Okabe

Hirokazu Okada

keisuke Okita

Hanna Oksanen

Satoru Okuda

Anthony Olarerin-George

Sandrine Ollagnier-de Choudens

Jesper Olsen

Bradley JSC Olson

Munenori Ono

Noriaki Ono

Timothy Opperman

Guy Orban

Matej Oresic

Caitlin Orsini

Javier Ortega-Hernandez

Karin Ortmayr

Michael Ostap

Masaki Otagiri

Daniel Otzen

Michael Overduin

Suat Özbek

Aydogan Ozcan

Andrew Ozga

Elif Ozkirimli

**Pabst—Perry**

Georg Pabst

Kevin Padian

Pablo Paez

Michele Pagano

Bhupinder Pal

Benoit Palancade

Oleg Paliy

Caroline Palmer

Lifeng Pan

Lin Pan

Xiaoyue Pan

Theofanis Panagiotaropoulos

Kaushik Panda

John Panepinto

Kristen Panfilio

Stanley Pang

Kostas Pantopoulos

Salvatore Papa

Efthymia Papaevangelou

Evangelia Papakonstanti

Irinna Papangeli

Bjoern Papke

Jordi Paps

Emilio Parisini

Eunsook Park

Kevin Park

Ki-Soo Park

Kyung-Soon Park

Woong-Yang Park

Hugo Parker

Simona Parrinello

Carrie Partch

Ben Pascoe

José-Carlos Pastor-Jimeno

Avinash Patel

Dhaval Patel

Gavin Paterson

Nitin Aniruddha Patil

Sushant Patil

Hirak Kumar Patra

Bill Patterson

Joshua Patterson

Perrine Paul-gilloteaux

Anirban Paul

Laurens Pauwels

Rachael Pearson

François Jean Peaudecerf

Stine Pedersen

Isabela Pedroza-Pacheco

Duanqing Pei

Yonggang Pei

Claudia Peitzsch

Thaher Pelaseyed

Jerry Pelletier

Nieves Peltzer

Francisco Peñagaricano

Alberto Pendas

Yong Peng

Fabio Penna

Pleuni Pennings

Gertrudis Perea

Francisco Pereira

Jesus Perez-Gil

David Perez-Gonzalez

Matthew Peros

Steve Perry

**Pestios—Putman**

Elizabeth Pestios

Elsa Petit

Michaela Petter

Sari Peura

Adrien Peyrache

Paul Pharoah

Katrin Philippar

Benjamin Philmus

Giulia Piaggio

Shilong Piao

Gary Piazza

Colette Lafontaine Picard

Kathryn Picard

Pietro Picconi

Simone Picelli

Nicole Piche-Nicholas

Sacha Pidot

Monica Piergiovanni

Alexander Pietras

Ruth Pietri

Hugh Piggins

Marc Pilon

Fabien Pinaud

Itziar Pinilla-Macau

Dimitris Pinotsis

Stefano Piraino

Mathias Mistretta Pires

Alexandros Pittis

Hilke Plassmann

Julie Plastino

Dietmar Plenz

Maksim Plikus

Joshua Plotkin

Sharon Pochron

Christine Podrini

Ehmke Pohl

Guy Poirier

William Polacheck

Lubos Polerecky

Igor Polikarpov

Martin Pollak

Kenneth Michael Pollard

Kornelia Polyak

Adrian Ponce Alvarez

Frederic Pontvianne

Kate Poole

Sorina Popescu

Juergen Popp

Eduard Porta

Klaas Martinus Pos

Danielle Posthuma

Georgios Pothoulakis

Gino Poulin

Mónica Pradillo

Veena Prahlad

Simant Prakoonwit

Peter Preiser

Rytis Prekeris

Tino Prell

Marco Presta

Jill Preston

Lynda Prior

Simone Proemel

Ludmila Prokunina-Olsson

Christopher Gregory Proud

Mihaela Pruteanu

Nikolaos Psonis

Holger Puchta

Joseph Puglisi

Nathan Putman

**Q**

Ting Qi

Chao-Nan Qian

Yi Qiao

Lidong Qin

Xiao-Bo Qiu

Lorna Quandt

Natalina Quarto

Tiago Quental

Romain Quentin

Ethel Queralt

Jason Quinn

**Raab—Roager**

Nancy Raab-Traub

Jeremy Van Raamsdonk

Grazia Daniela Raffa

Mehran Rahimi

Pasquale Raia

Gregor Rainer

Nagarajan Raju

Markus Ralser

Suresh Ramakrishna

Chongzhao Ran

Rohan Ranasinghe

Jayne Raper

Leda Raptis

Mark Rasenick

Gordana Rašić

Sepand Rastegar

Catherine Ravel

Maddaly Ravi

J. Christian Ray

Julie Rayes

Julian Rayner

Stephen Rayport

Sai Reddy

Sarah Reece

Aviv Regev

Andrew Reid

Alan Remaley

Xiaojun Ren

André Figueiredo Rendeiro

Nicolas Renier

Christopher Rensing

Scott Retterer

Bruno Reversade

Alba Rey-Iglesia

Adrian Reyes-Prieto

William Rice

Jeremy Rich

Peter Richards

Tom Richards

Lynn L Richardson

Rodney Richardson

Sandra Richardson

Sara Richter

Anna Rising

Olivia Rissland

Brendan Ritchie

Ciro Rivera-Casas

I. Ortiz Rivera

Julio Rivera

Silvio Rizzoli

Henrik Roager

**Roberts—Ryan**

James Roberts

Alison Robertson

Claire Robertson

Robert Robey

Harlan Robins

Marc Robinson-Rechavi

Jean-David Rochaix

Alvaro Rodriguez

Ryan Roeder

Fabio Romerio

Vittoria Roncalli

Laure Rondi-Reig

Shisong Rong

Christopher Rongo

Jeanne Ropars

Rui Rosa

German Rosano

Evan Rosen

Yaeli Rosenberg

Leah Rosin

Zuzanna Maria Rosin

Hannes Rost

Siegfried Roth

Gideon Rothschild

Tracey Rouault

Nicolas Rouhier

Kyle Roux

Lajos Rozsa

Beibei Ru

Karolina Rudnicka

Rüdiger Rudolf

Francesco Simone Ruggeri

Ynte Ruigrok

J.C. Ruiz-Suárez

Natividad Ruiz

Joseph Runde

Heather Ruskin

Mark William Russell

Błażej Ruszczycki

Marcello Ruta

Timothy Ryan

**Sabate—Schröder**

Raimon Sabate

Frederick Sachs

Kyle Saeterstrom

Yvan Saeys

Stephen Safe

Robert Safirstein

Alvaro Sagasti

Pankaj Sah

Suvrajit Saha

Jason Sahl

Susmita Sahoo

Laura Säisänen

Shuzo Sakata

Tetsushi Sakuma

Kunihiko Sakumi

Hiroaki Sakurai

Roberto Parra Saldívar

Mohammed Saleem

Hamideh Salehi

Paolo Salomoni

Arneet Saltzman

Jason Samaha

Harini Sampath

Mikko Sams

Hugo Sanabria

Hector Sánchez

Goar Sanchez-Sanz

Teresa Sanchez

Geir Kjetil Sandve

Joshua Sanes

Serena Sanna

Raffaella Santoro

Subhabrata Sanyal

Suparna Sarkar-Banerjee

Abhishek Sarkar

Daisuke Sato

Noriyuki Satoh

Sandro Satta

Rita Sattler

Philippa Sauders

Jeanne Savage

Rebecca Saxe

Nazish Sayed

Suzie Scales

Marcel M Schaaf

Erik Schäffer

Paul Schanda

Richard Scheltema

Nico Scherf

Ueli Schibler

Menno Schilthuizen

David Schlaepfer

Marie-Lise Schlappy

Helgo Schmidt

Mathias Schmidt

Miriam Schmidts

Igor Schneider

Sylvie Schneider

Jana Schniete

Stephen Schoenberger

Bob Scholte

Marieke Schölvinck

Glen Scholz

C. Mary Schooling

Michael Schroda

Gunnar Schröder

**Schubert—Shu**

Wolf-Dieter Schubert

Frederik Schulz

Fredrick Ray Schumacher

Volker Schünemann

Erwin Schurr

Juan Manuel Schvartzman

Yannick Schwab

Denis Schwartz

Russell Schwartz

Edward Schwarz

Roland Ernst Schwarzenbacher

Katerina Schwarzerova

François Schweisguth

Michael Scott

Sara Sdelci

Matthew Seaman

Dorothy Sears

Arnau Sebé-Pedrós

Adriano Sebollela

Markus Seeger

Esha Sehanobish

Shiori Sekine

Jorge Sepulcre

Edgardo Sepulveda

Tiago Serafim

Melody Serena

Vahid Serpooshan

Thomas Serre

Jaswinder Sethi

Claire Sexton

Mathew Seymour

Eleonora Sforza

Sergey Shabala

Ivan Shabo

Yousif Shamoo

Hongyan Shan

Weixing Shan

Rebecca Shansky

Fangwei Shao

Jian-zhong Shao

Robert Shapley

Shalini Sharma

Dalal Shawky

Vasu Sheeba

Anantha Shekhar

Medha Shekhar

Anna Shen

Gaozhong Shen

Guochun Shen

Michael Shen

Tang-Long Shen

Jianlin Shi

Jianxin Shi

Jianxin Shi

Shuo Shi

Xiaobing Shi

Yufei Shi

Ayala Shiber

Sang-Hee Shim

James Shine

Yoshitsugu Shiro

Nikolay Shirokikh

Yuichiro Shirota

Maya Shmulevitz

Matthew Shoulders

Dilip Shrestha

Xiaokun Shu

**Shulman—Starkl**

Gordon Shulman

Kimberly Siegmund

Ella Tali Sieradzki

Sara Sigismund

Virginijus Siksnys

Jonathan Silberg

Christian Siltanen

Roberto Silva

Juha Silvanto

Daniele Silvestro

Fraser James Sim

Lukas Simon

David Sinclair

Charlotte Sinding

Manvendra Singh

Sivaraj Sivaramakrishnan

Heather Smallwood

Younes Smani

Age Smilde

Daniel Smith-Paredes

Aaron Smith

Andrew Michael Smith

Ann Smith

C Wayne Smith

Chris Smith

Cody Smith

Stacey Smith

Thomas Smith

Sander Smits

Emilie Snell-Rood

Hans-Willem Snoeck

Ruben Soares

Robert Sobol

Carolina Soekmadji

Olga Sokolova

Susana Solá

Shyam Solanki

Ken Solt

Alice Soragni

Federica Sotgia

Doug Speed

Paul Spellman

Lisa Spencer

Thomas Spencer

Felipe Sperandio

Ziv Spiegelman

Katherine Spindler

Stefania Spranger

Thomas Spratt

Pirjo Spuul

Martin St Maurice

Charlotte Stagg

Claire Stanley

Daphne Stapels

W. Marshall Stark

Philipp Starkl

**Starko—Szabo**

Sam Starko

Diana Stavreva

David Steadman

Jan Steinkühler

Jevrosima Stevanovic

Martin Stevens

Nicola Stevens

Rachel Stine

Martin Stoffel

Darko Stojkov

Andreas Stolz

Tim Storr

Rolf Stottmann

Peter Stougaard

Josh Strable

Mathew Stracy

Henrik Strahl

Timothy Strauman

Sigmar Stricker

Luke Strotz

Aaron Frank Struck

Devi Stuart-Fox

Dennis Stuehr

Jorg Stulke

Nicholas Stylopoulos

Xin-zhuan Su

Elavarasan Subramani

Karthik Subramanian

Narasimhan Sudarsan

Yukihiko Sugimoto

Karsten Suhre

Roger Summons

feng sun

Jennifer Sun

Jinpeng Sun

Jun Sun

Qiang Sun

Yujie Sun

Jennifer Sunday

Cathryn Sundback

Ching-Hwa Sung

Sibum Sung

Ida Surakka

M. Azim Surani

Dawid Surmik

Richard Sutton

Mario Luca Suva

Madoka Suzuki

Osamu Suzuki

Maksim Svetlov

Matt Swulius

Lorraine Symington

Tatyana Sysoeva

Amanda Szabo-Reed

**Tabassum—Thompson**

Sartaj Tabassum

Enzo Tagliazucchi

Hidetoshi Tahara

Akira Tajima-Goto

Junji Takabayashi

Hidenori Takahashi

Masahide Takahashi

Hirotomo Takatsuka

Tetsuo Takehara

Yoshinori Takei

Yuen Yi Tam

Arielle Tambini

Marco Tamietto

Robert Tampé

Giedre Tamulaitiene

Shyh-Han Tan

Dean Tang

Toshiyasu Taniguchi

Julian Alexander Tanner

Jianning Tao

Wei Tao

Justin Taraska

Ann Tarrant

Gian Gaetano Tartaglia

Eric Tartour

Yvonne Tay

Aimee Taylor

Alex Taylor

James Taylor

Jonathan Taylor

Jordan Taylor

Hein te Riele

Shunsuke Tei

Amalio Telenti

Priscila Tempaku

Hanna ten Brink

Joshua Tenenbaum

Stefan Terjung

Andrew Teschendorff

David Thal

Robert Thänert

Michael Theisen

Michel Thiebaut de Schotten

Axel Thielscher

Bram Thijssen

Gary Thomas

Jason Thomas

Fabiano Thompson

Michael Thompson

Nathan Thompson

**Thomsen—Tzingounisz**

Hauke Thomsen

Doreen Thor

Andrew Thorburn

Harry Thorpe

Gregor Thut

Summer Thyme

Yang Tielin

Ruth Timme

Joanna Timmins

Richard Ting

Zakir Tnimov

David Tolhurst

Iva Tolic

Giovanna Tomaiuolo

Lubomir Tomaska

Michael Tomasson

Tatsuya Tomo

Jurriaan Ton

Timothy Topper

Davis Torrejon

Kalman Tory

Zlatko Trajanoski

Tatjana Trceku

Xavier Trepat

Kyle Tretina

Adrian Treves

Juan Luis Trincado Alonso

Timir Tripathi

Eirini Trompouki

George Truskey

Francis Tsai

Aristidis Tsatsakis

Atsushi Tsuda

Moriya Tsuji

Yoshiaki Tsuji

Vassiliy Tsytsarev

Budd Tucker

Danielle Tullman-Ercek

Margherita Yayoi Turco

Kürsad Turgay

Robert Turner

Robert Tycko

Anna Tyler

Jeffrey Wallace Tyner

Esa Tyystjärvi

Anastasios Tzingounisz

**U**

Seichii Uchiyama

Joshua Udall

Severin Uebbing

Hiroki Ueda

Lene Uhrbom

Hidemitsu Uno

Caitlin Uren

Samuel Urmy

Arto Urtti

Yuri Ushkaryov

Barbara Uszczyńska-Ratajczak

**V**

Thomas Vaccari

Gerben Vader

Bharat Vaidyanathan

Ioannis Vakonakis

Hector Valdivia

Antoni Valero-Cabré

Daniela Vallone

Leo Valon

Daniël Van Damme

Alex Van der Bliek

Ingrid van der Laan-Luijkx

Silvère van der Maarel

Philip Anton van der Merwe

Bas van der Schootbrugge

Allen Van Deynze

Schuyler van Engelenburg

Ronald van Kesteren

Frank Johannes Maria van Kuppeveld

Evert van Nieuwenburg

Koen Van Rompay

Sven van Teeffelen

Lodewijk van Walraven

Robert VanBuren

Matthew Vander Heiden

Jean Vannier

Alejandro Vaquero García

Jobin Varkey

Arul Varman

Ajith Vasanthakumar

Angélica da Silva Vasconcellos

Carlo Vascotto

Leva Vasiliauskaité-Brooks

Athanasios Vasilopoulos

Roel Veerkamp

Akos Vegvari

Niels Verhulst

Louis Vermeulen

Elisabetta Versace

Chris Verschoor

Ales Vetesnik

Vaiva Vezys

Daniel Vidal

Felipe Vieira Braga

Martin Viklund

John Viles

Agnese Viliuma

Carlos Villalobos

Jane Visvader

Panagiotis Vlachostergios

Dennis Voelker

Adam Vogel

Matthias Christian Vogg

Thomas Vogl

Fabian Voigt

Irina Voineagu

Sinisa Volarevic

David Volk

Mirko Völkers

Lucienne Vonk

Logan James Voss

Karen Vousden

**Wacker—Werner**

Daniel Wacker

allison walker

Mark Edwin Walton

S Patrick Walton

Matthew Wanat

Brian Wandell

Andrew Wang

Chung-Ju Rachel Wang

Congli Wang

Fan Wang

Guirong Wang

Hangxiang Wang

Hong Wang

Hua Wang

Junwen (John) Wang

Kaile Wang

kankan wang

Liang Wang

Lizhong Wang

Ming-Wei Wang

Peipei Wang

Tim Wang

Tobias Wang

W Wang

Wang Wang

Wen-Ching Wang

Xiaoming Wang

Xingxing Wang

Xiuxing Wang

Xuefeng Wang

Xusheng Wang

Yang Wang

Yi Wang

Zhanhui Wang

Lucas Daniel Ward

Sally Ward

Gary Warnes

Steven Wasserman

Satoshi Watanabe

Michael Waters

Christine Watson

Ashley Webb

Eric Webb

Gert Weber

Richard Webster

Scott Webster

Robbee Wedow

Harshini Weerasinghe

Susanne Wegmann

Guowei Wei

Wenyi Wei

Uwe Weierstall

Eric Weimer

Michael Weiner

Gordon Weir

William Weis

Lucien Weiss

Cornelia Welte

Quan Wen

Xianghua Wen

Krister Wennerberg

Theodore Wensel

Donna Werling

Christian Werner

**Wertheim—Wytock**

Bregje Wertheim

Niels Wessel

Daniel Wesson

Emma West

Michelle Joanne West

Benedikt Westermann

E. John Wherry

Alan White

Brian White

Andrew Whitehead

Carolin Wichmann

Ryan Robert Wick

Reed Wickner

Imam Widhiono

Joerg Wiedenmann

André Barretto Bruno Wilke

Eric Wilkey

Holger Wille

Cory Williams

Sylvain Williams

Matthew Wills

Donald Wilson

Hugh Wilson

Mark Wilson

William Wilson

Ian Windsor

Craig Winstanley

Maureen Wirschell

Aaron Wirsing

R. Luke Wiseman

Robert Wisse

Elizabeth Wohlfert

Martijn Wokke

Francis Wolenski

Mariana Federica Wolfner

Thilo Womelsdorf

You-Yeon Won

Ambroise Wonkam

Tonia Woodberry

Kerry Woods

Holly Noelle Woodward

Han Wosten

Richard Woychik

Gregory Wray

Gerard Wright

Baojian Wu

Chengbiao Wu

Guojun Wu

Jianyu Wu

Po-Hung Wu

Ronghu Wu

Shuang Wu

Zhijian Wu

Gerhard Wunderlich

Thomas Wytock

**X**

Iro Xenidou-Dervou

Zhiyong Xi

Jianyang Xia

Yuqiong Xia

Jianbin Xian

Han Xiao

Jun Xiao

Yinghong Xiao

Linglin Xie

Zijian Xie

Da Xing

Yong Xiong

Jie Xu

Lifeng Xu

Ping Xu

Pinglong Xu

Xiaodong Xu

Youwei Xu

Yunyao Xu

Wei-Feng Xue

Yansong Xue

**Y**

Norikazu Yabuta

Gaya Prasad Yadav

Hiroshi Yagi

Mamiko Yajima

Alexander Yakunin

Mehmet Yalvac

Ryosuke Yamada

Takahiro Yamashita

Qingpi Yan

Yajun Yan

María Yáñez-Mó

Bao Yang

Chengwei Yang

Chun Yang

Dahai Yang

Dongping Yang

Fentang Yang

Jian Yang

Jinshui Yang

Samuel Yang

Yingzi Yang

Robert Yarchoan

Ryohei Yasuda

Andrew Yates

Masayuki Yazawa

Bang-Ce Ye

Jianping Ye

Todd Yeates

Joseph Yeeles

Edward T.H. Yeh

Pamela Yeh

Jiun Yen

Chengqi Yi

Feng Yi

Zhitong Yin

Anne Yoder

Koichi Yoneyama

Young-sup Yoon

Lingchong You

Eric Young

Janet Young

Jason Young

Ji Young

Ihab Younis

Edward Yu

Eun Young Yu

Hongwei Yu

Jianping Yu

Kebing Yu

Quan-You Yu

Xiaochun Yu

Guo-cheng Yuan

Xunlai Yuan

Yao-Wu Yuan

Natalya Yutin

**Z**

Stephanie Zaffran

Francisco Zafra

Peter Zandstra

Theodore Zanto

Ryan Zapotocny

Roberta Zappasodi

Lynn Zechiedrich

Aleksej Zelezniak

Ling-Li Zeng

Wenwen Zeng

Gabriel Etienne Zentner

Branko Zevnik

Dabing Zhang

Ge Zhang

Gongyi Zhang

Hongkai Zhang

Jian-Ting Zhang

Jianwei Zhang

Jianxiang Zhang

Jin Zhang

Kang Zhang

Lei Zhang

Li Zhang

Liangsheng Zhang

Libing Zhang

Linlin Zhang

Peijing Zhang

Shihua Zhang

Thianzhen Zhang

Weizhen Zhang

Weizhou Zhang

Wen Zhang

Xiaowei Zhang

Yijing Zhang

Yu Zhang

Zhao Zhang

Zhihao Zhang

Chunzhao Zhao

Huaying Zhao

Meiping Zhao

Yanxiang Zhao

Yun Zhao

Jie Zheng

Wenyi Zheng

Feng-Quan Zhou

Guang-Biao Zhou

Guangbin Zhou

Qi Zhou

Wenbo Zhou

Yongfeng Zhou

Zhong‐Wei Zhou

Banghe Zhu

Danmeng Zhu

Jian-Kang Zhu

Longfu Zhu

Ping Zhu

Yingjie Zhu

Victor Zhurkin

Danila Zimenkov

Christophe Zimmer

Manuel Zimmer

Wolfram Zimmermann

Alessio Zippo

Eric Zollars

Chenghang Chuck Zong

Katerina Zorina-Lichtenwalter

Qiang Zou

Quan Zou

Chloe Zubieta

Andrea Zuccolo

Lorena Zuliani-Alvarez

Paul Zwack

